# Mapping the Philippines’ Mangrove Forests Using Landsat Imagery

**DOI:** 10.3390/s110302972

**Published:** 2011-03-07

**Authors:** Jordan B. Long, Chandra Giri

**Affiliations:** 1 US Geological Survey, Earth Resources Observation and Science Center (EROS), Sioux Falls, SD 57198, USA; E-Mail: jlong@usgs.gov; Tel.: 605-594-2903; 2 ARSC Research and Technology Solutions, contractor to U.S. Geological Survey (USGS) Earth Resources Observation and Science Center, Sioux Falls, SD 57198, USA

**Keywords:** mangrove, Landsat, mapping, unsupervised classification

## Abstract

Current, accurate, and reliable information on the areal extent and spatial distribution of mangrove forests in the Philippines is limited. Previous estimates of mangrove extent do not illustrate the spatial distribution for the entire country. This study, part of a global assessment of mangrove dynamics, mapped the spatial distribution and areal extent of the Philippines’ mangroves circa 2000. We used publicly available Landsat data acquired primarily from the Global Land Survey to map the total extent and spatial distribution. ISODATA clustering, an unsupervised classification technique, was applied to 61 Landsat images. Statistical analysis indicates the total area of mangrove forest cover was approximately 256,185 hectares circa 2000 with overall classification accuracy of 96.6% and a kappa coefficient of 0.926. These results differ substantially from most recent estimates of mangrove area in the Philippines. The results of this study may assist the decision making processes for rehabilitation and conservation efforts that are currently needed to protect and restore the Philippines’ degraded mangrove forests.

## 1. Introduction

Mangroves are salt tolerant trees and shrubs that grow within the sheltered marine intertidal zones of the tropics and subtropics. As a whole community, mangroves are capable of thriving in a wide range of harsh environmental conditions and share unique adaptive traits such as salt excreting leaves, exposed breathing root system, and production of viviparous propagules [1]. The Philippines’ mangrove forests offer numerous ecosystem goods and services to coastal populations. Mangrove is traditionally used for firewood, charcoal, alcohol, medicines, and thatching used for construction [2,3]. Furthermore, these forests provide vital ecological services such as bioprotection from coastal erosion [4], nursery and feeding sites for marine species [5], and the possible reduction of the devastating impacts of tropical storms and tsunamis [6,7].

Human activities, however, have altered much of the mangrove forests within the Philippines over the past century. The total mangrove area in the Philippines has decreased by almost half [8], from an estimated 500,000 ha in 1918 [2]. A major driving force of mangrove forests loss in Southeast Asia, and in the Philippines, is the rapid expansion of aquaculture development [9]. Within the Philippines alone, an estimated 50 percent of mangrove deforestation can be directly attributed to brackish-water pond development [10]. Mangrove degradation in the Philippines is anticipated to continue [11], despite greater conservation and localized replanting efforts [12].

The objective of this study is to accurately quantify mangrove areal extent and map the spatial distribution of mangrove forests in the Philippines. The unbiased data generated may be used as a reliable measurement for monitoring future changes in the Philippines’ mangrove forests.

## 2. Study Area

**Figure 1.**
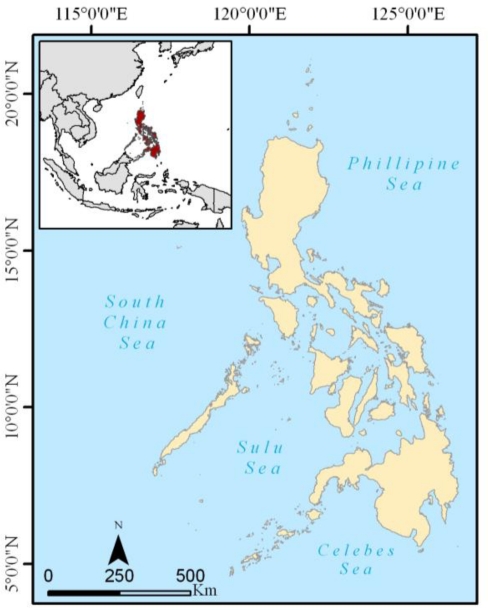
Location of study area.

The Philippines is an archipelago made up of 7,107 islands located completely within the tropics off the southeastern coast of Asia (Figure 1). The terrain is mostly mountainous with narrow to extensive coastline. The coastline extends 36,289 km and is surrounded by the waters of the Celebes and Sulu Seas along its southern coast, the South China Sea along the western coast, and the Philippine Sea along its eastern coast.

The Philippine islands are considered one of the top biodiversity “hot spot” areas of the world, supporting 1.9 percent of the world’s endemic plants and vertebrate species [13]. Mangrove diversity is relatively high in the Philippines with 35 true mangrove species compared with North and Central America, which combined have 10 species. Only Indonesia (43), Malaysia (41), Australia (37), and Papua New Guinea (37) have greater mangrove biodiversity than the Philippines [14].

## 3. Data and Methodology

### 3.1. Data

**Figure 2.**
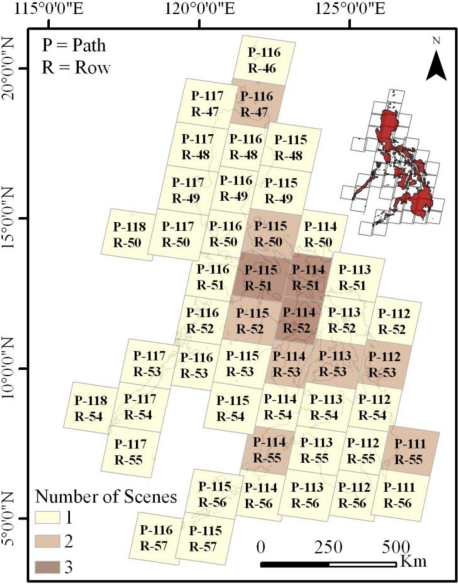
Number of Landsat scenes required for cloud-free classification per path and row.

**Figure 3.**
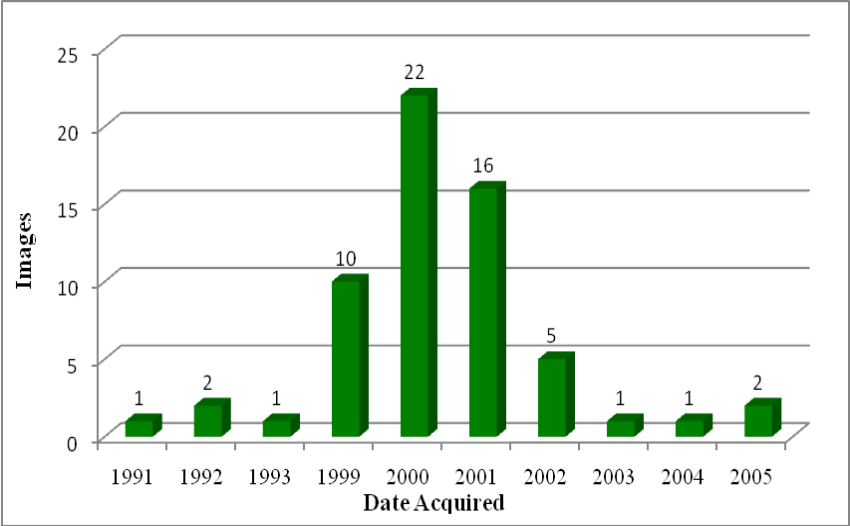
Majority of imagery used for analysis were acquired circa 2000.

We primarily used publicly available Global Land Survey (GLS) 2000 Landsat 30-meter resolution data to map the Philippines’ mangrove extent and spatial distribution for the year circa 2000. These data were acquired from the U.S. Geological Survey (USGS) Earth Resources Observation and Science Center (http://glovis.usgs.gov). Global Land Survey (GLS) 2000 data were processed using existing GeoCover and Landsat archive to produce a near-global, cloud-free mosaic [15]. Ideally, only GLS 2000 data would have been used to map mangrove spatial distribution and areal extent for the year 2000. However, because of persistent cloud cover in some areas, multiple images were intermittently required to classify a single path and row (Figure 2). Therefore, data from GLS 1990, GLS 2005, and the Landsat archive were occasionally substituted when cloud free GLS 2000 imagery was unavailable (Figure 3). In total, 61 Landsat images were required to classify 47 Landsat paths and rows.

### 3.2. Methods

Prior to classification, bands 1, 2, 3, 4, 5, and 7 were stacked into a composite image and were subsequently masked to include only areas where mangrove forest is likely to occur (*i.e.*, low-lying coastal areas and intertidal zones) and to exclude areas where mangrove forest is not naturally located (*i.e.*, far inland, highlands, and open ocean). Masking an area of interest reduces data volume and increases overall classification accuracy by reducing the amount of land cover types and spectral variation [16].

Following masking, images were classified using an Iterative Self-Organizing Data Analysis Techniques (ISODATA) algorithm. An ISODATA algorithm requires the user to choose the initial estimates of class means, and then each pixel is assigned to classes with a similar mean; in this respect, ISODATA resembles an unsupervised classification [17]. The process of assigning pixels to a class is repeated until reaching the maximum number of iterations set by the user.

**Figure 4.**
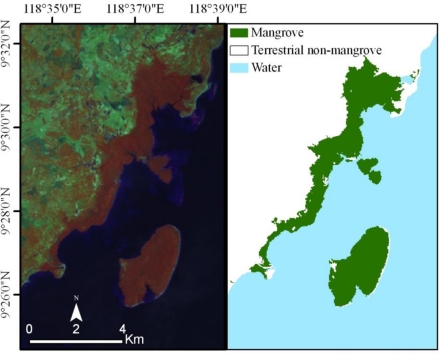
Landsat satellite image including mangrove forest cover (**left**) on Palawan Island, Philippines, compared with classification results (**right**). Intact mangrove forest is relatively distinct and appears bright orange in false color band combination 4, 5, and 7 (left).

For our study, 100 to 150 clusters were generated using 15 maximum iterations and a convergence threshold of 0.950. Through manual interpretation, clusters were merged into three classes based on spectral similarities: mangrove, terrestrial non-mangrove, and water. Following classification and clustering, additional recoding was performed to eliminate apparent classification errors. Figure 4 illustrates a false color Landsat image containing mangrove forest and exemplifies the classification results after additional recoding was performed.

Following reclassification of all imagery, a mosaic of the entire country was prepared. A gap analysis was performed by comparing the national mosaic with all original Landsat imagery to ensure no mangrove areas were missed. An accuracy assessment was conducted using a total of 150 stratified random points: 100 random points for mangrove classified areas and 50 random points for terrestrial non-mangrove classified areas. The randomly generated classified points were then compared with ancillary data such as land cover maps, Google Earth™, and high-resolution satellite data from GeoEye to verify land cover classification accuracy.

## 4. Results and Discussion

**Figure 5.**
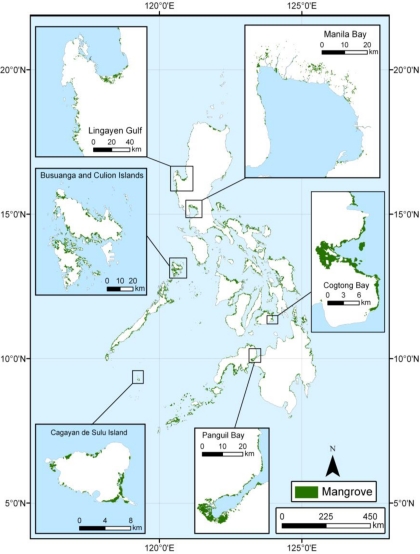
Spatial distribution of mangrove forests of the Philippines for 2000.

**Figure 6.**
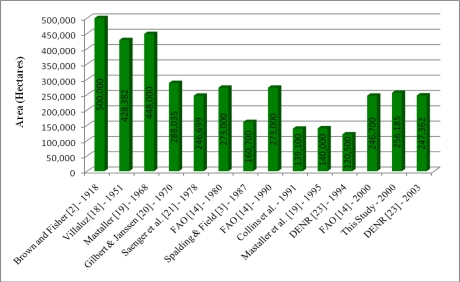
Comparison of areal estimates of mangrove forest for the Philippines. Dates indicate year of estimate.

We prepared a wall-to-wall map of mangrove extent and spatial distribution of the entire country of the Philippines (Figure 5). With this new mangrove database of the Philippines, we delineated the spatial distribution and assessed the areal extent of mangrove forests at a national scale. We estimated that total area of the Philippines’ mangrove forests was 256,185 ha. circa 2000. Our national assessment is marginally higher than the most recent estimates published by the FAO [14] and the Philippine Department of Environment and Natural Recourses (DENR) [23] (Figure 6). These estimates were produced through differing methods and technologies. The FAO utilized “reliable” estimates from previously published and unpublished sources to calculate the mangrove extent for 2000. The DENR 2003 estimate was derived from interpretation of 2001–2003 Landsat imagery. This analysis, however, was part of a broad national land cover mapping project, which could have resulted in an underestimation of mangrove areal extent due to a higher occurrence of misclassification.

Similar to the DENR, this study used 30-meter moderate resolution imagery, that can map small patches (0.009 ha or greater) of mangrove; however, we only mapped three land cover classes and primarily focused on mapping of mangrove forests. Although it was not possible to solely use Landsat imagery acquired in 2000 because of atmospheric contamination, the majority of imagery used in our analysis was acquired circa 2000.

**Figure 7.**
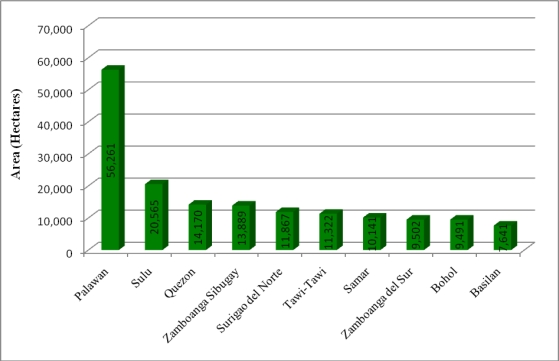
Provinces with majority of mangrove extent.

The results of an accuracy assessment indicate a high rate of classification accuracy with a user accuracy of 95% for mapping mangrove forest and 100% for terrestrial non-mangrove areas, an overall accuracy of 96.6%, and a kappa coefficient of 0.926. Our results revealed that 66 out of the Philippines’ 82 provinces contained mangrove (Table 1), with the largest areas of mangrove forests located on the island provinces of Palawan and Sulu (Figure 7).

**Figure 8.**
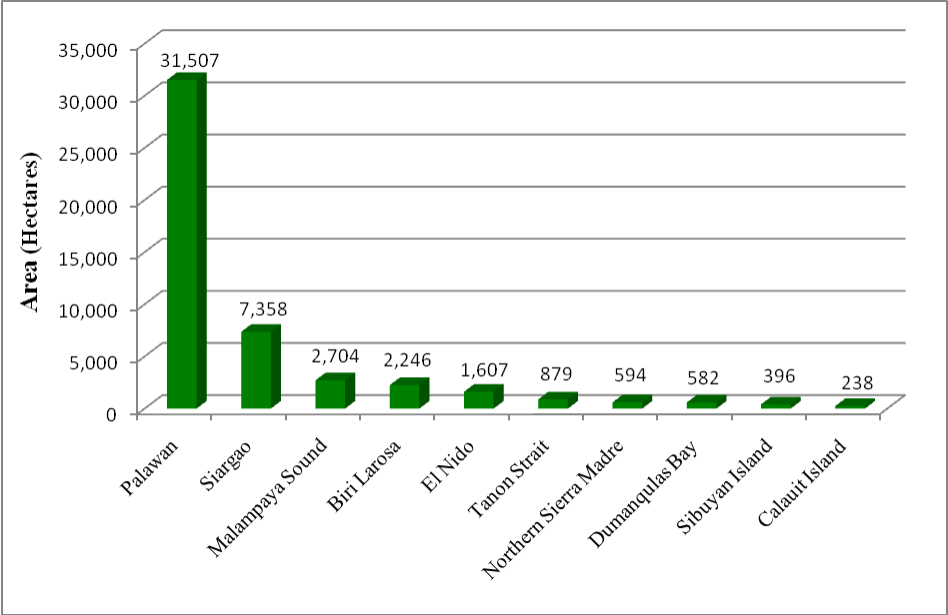
Mangrove protected aeas under IUCN delineated protected areas I–VI.

Notably, 19% (49,363 ha) of the Philippines’ total mangrove area is located within existing protected area networks (International Union for Conservation of Nature (IUCN) protected areas categories, I-VI), with the greatest area of protected mangroves located on Palawan (Figure 8). The IUCN declares a protected area as “A clearly defined geographical space, recognized, dedicated and managed, through legal or other effective means, to achieve the long-term conservation of nature with associated ecosystem services and cultural values [24]”. The IUCN protected area management categories are an important global standard for the planning, establishment, and management of protected areas.

Although this study provides a detailed analysis of the areal extent and distribution of the Philippines’ mangrove forests, more qualitative and quantitative information concerning the quality and condition of mangrove forest is needed for scientific planning and conservation efforts in the Philippines.

## 5. Conclusions

Based on our analysis that used remotely sensed satellite observations and digital image classification techniques, our investigation offers a high resolution and comprehensive mapping of mangrove for the Philippines. These data are reliable measurements and may be referenced to assess future changes in the Philippines’ mangrove forests. Furthermore, the results of this study may assist the decision making processes for rehabilitation and conservation efforts that are needed to protect and restore the Philippines’ degraded mangrove forests. Future analysis of the Philippines’ mangrove forests will be required to determine the status and trends of mangrove dynamics. Possible scenarios of mangrove degradation, deforestation, and redistribution (including the effects of rapid sea level rise) may be modeled using geospatial data generated by this study.

## Figures and Tables

**Table 1.** Mangrove areal extent by province.

**Province**	**Area (Hectares)**	**National Percentage**	**Province**	**Area (Hectares)**	**National Percentage**
Agusan del Norte	244.98	0.1	Leyte	5,807.07	2.26
Aklan	1,144.44	0.45	Maguindanao	907.92	0.35
Albay	1,081.17	0.42	Marinduque	2,732.22	1.06
Antique	945.9	0.37	Masbate	5,302.08	2.06
Aurora	497.07	0.19	Metropolitan Manila	39.69	0.02
Basilan	7,641.18	2.97	Misamis Occidental	2,066.49	0.8
Bataan	238.59	0.09	Misamis Oriental	341.19	0.13
Batangas	508.95	0.2	Negros Occidental	4,393.26	1.71
Biliran	231.39	0.09	Negros Oriental	2,004.93	0.78
Bohol	9490.5	3.69	Northern Samar	4,286.52	1.67
Bulacan	391.14	0.15	Occidental Mindoro	1,842.93	0.72
Cagayan	5,175.27	2.01	Oriental Mindoro	2,975.31	1.16
Camarines Norte	3,628.17	1.41	Palawan	5,6261.3	22.23
Camarines Sur	5,315.31	2.07	Pampanga	251.73	0.1
Camiguin	4.95	0	Pangasinan	1,206.63	0.47
Capiz	1,999.8	0.78	Quezon	14,170	5.51
Catanduanes	1,671.3	0.65	Romblon	792.45	0.31
Cavite	35.73	0.01	Samar	10,140.6	3.94
Cebu	2,893.77	1.13	Sarangani	92.61	0.04
Compostela Valley	130.14	0.05	Shariff Kabunsuan	1,018.89	0.4
Davao del Norte	195.57	0.08	Siquijor	70.2	0.03
Davao del Sur	361.53	0.14	Sorsogon	3,895.74	1.52
Davao Oriental	1975.5	0.77	South Cotabato	13.86	0.01
Dinagat Islands	1654.56	0.64	Southern Leyte	643.68	0.25
Eastern Samar	5,595.93	2.18	Sultan Kudarat	949.95	0.37
Guimaras	577.08	0.22	Sulu	2,0564.8	8
Ilocos Norte	127.53	0.05	Surigao del Norte	11867	4.62
Ilocos Sur	228.87	0.09	Surigao del Sur	5,642.55	2.19
Iloilo	1,322.91	0.51	Tawi-Tawi	11,322.2	4.4
Isabela	592.29	0.23	Zambales	981.54	0.38
La Union	144.18	0.06	Zamboanga del Norte	1,961.82	0.76
Lanao del Norte	1,580.94	0.61	Zamboanga del Sur	9,501.66	3.7
Lanao del Sur	620.37	0.24	Zamboanga Sibugay	13,889.2	5.4
